# Chimeric Virus-like Particles of Physalis Mottle Virus as Carriers of M2e Peptides of Influenza a Virus

**DOI:** 10.3390/v16111802

**Published:** 2024-11-20

**Authors:** Elena A. Blokhina, Eugenia S. Mardanova, Anna A. Zykova, Marina A. Shuklina, Liudmila A. Stepanova, Liudmila M. Tsybalova, Nikolai V. Ravin

**Affiliations:** 1 Institute of Bioengineering, Research Center of Biotechnology of the Russian Academy of Sciences, 119071 Moscow, Russia; 2 Smorodintsev Research Institute of Influenza, Russian Ministry of Health, 197376 St. Petersburg, Russia

**Keywords:** Physalis mottle virus, virus-like particle, influenza A virus, M2e peptide, vaccine

## Abstract

Plant viruses and virus-like particles (VLPs) are safe for mammals and can be used as a carrier/platform for the presentation of foreign antigens in vaccine development. The aim of this study was to use the coat protein (CP) of Physalis mottle virus (PhMV) as a carrier to display the extracellular domain of the transmembrane protein M2 of influenza A virus (M2e). M2e is a highly conserved antigen, but to induce an effective immune response it must be linked to an adjuvant or carrier VLP. Four tandem copies of M2e were either fused to the N-terminus of the full-length PhMV CP or replaced the 43 N-terminal amino acids of the PhMV CP. Only the first fusion protein was successfully expressed in *Escherichia coli*, where it self-assembled into spherical VLPs of about 30 nm in size. The particles were efficiently recognized by anti-M2e antibodies, indicating that the M2e peptides were exposed on the surface. Subcutaneous immunization of mice with VLPs carrying four copies of M2e induced high levels of M2e-specific IgG antibodies in serum and protected animals from a lethal influenza A virus challenge. Therefore, PhMV particles carrying M2e peptides may become useful research tools for the development of recombinant influenza vaccines.

## 1. Introduction

Virus-like particles (VLPs), structurally similar to viruses, are among the most effective and frequently used carriers. VLPs can be formed as a result of the spontaneous assembly of viral capsid proteins into high-molecular structures resembling the parent virus but without the viral genome. The structural features of VLPs make them attractive platforms for the development of recombinant vaccines, primarily against viral diseases (reviewed in [1]). VLPs mimic the corresponding viruses in size (usually 20–200 nm), spatial structure, and the ability to induce T-helper cells following the phagocytosis and processing of VLPs by antigen-presenting cells [2]. The secretion of cytokines by activated T-helper cells stimulates other immune cells, such as macrophages, B cells, and T cells [2]. In addition, due to the repetitive structure and the presence of B-cell epitopes, VLPs are actively recognized by B-cell receptors and induce a B-cell immune response [3]. The use of VLPs is safe because they lack the viral, infectious genetic material and are unable to revert to an infectious form.

VLPs can be used not only to protect humans and animals against the virus from which they are derived but also to display heterologous antigens of human and animal viruses on the surface of chimeric VLPs in a repetitive manner [1]. The purpose of chimeric VLPs is to induce immune responses against heterologous antigens displayed on the VLPs. Although VLPs based on the capsid proteins of human and animal viruses are most often used for the presentation of foreign antigens, VLPs from other organisms (including plant and bacterial viruses) can also be used to develop chimeric VLPs.

Plant viruses and VLPs carrying foreign antigens are powerful vaccine components due to their highly organized, repetitive spatial structure [4]. In fact, Tobacco Mosaic Virus particles, which display poliovirus epitopes on their surface, were one of the first examples of chimeric VLPs used for vaccine development [5]. Subsequently, VLPs of many plant viruses, both spherical and rod-shaped [6], were used as carriers of various antigens [4,7]. To present target antigens on the surface of VLPs formed by the capsid proteins of plant viruses, both genetic methods (creation of a gene encoding a capsid–antigen fusion protein) and chemical or enzymatic conjugation of the antigen with unmodified VLPs can be used [4]. In most studies, plant viral VLPs were obtained in plant expression systems or animal cells. However, bacterial expression remains the simplest and least expensive system for producing recombinant proteins. The capsid proteins of a number of plant viruses have been obtained in *Escherichia coli*, including Cowpea Chlorotic Mottle Virus [8], Johnson Grass Mosaic Virus [9], Physalis Mottle Virus [10], Papaya Mosaic Virus [11], Cardamom Mosaic Virus [12], and Potato Virus Y [13].

However, when choosing capsid proteins as carriers of antigens, spherical plant viruses with icosahedral symmetry seem to be the most promising since they have a more rigid structure, which is important for the further standardization of VLP-based vaccines. They also better mimic the structure of most viruses that cause infections in humans and animals. Of the listed viruses, only Cowpea Chlorotic Mottle Virus (CCMV) and Physalis Mottle Virus (PhMV) have spherical capsids. VLPs based on CCMV and PhMV have been used as antigen carriers [14,15,16,17].

PhMV is a single-stranded RNA virus. Its spherical capsid, composed of 180 subunits, has icosahedral T = 3 symmetry [18]. The PhMV capsid protein is well expressed in *E. coli* and assembled into VLPs, making it an attractive antigen carrier. The first studies used a method of genetically fusing the antigen to the CP. For this purpose, the N-terminus of the CP appears to be promising, since, unlike the C-terminus, it does not play a significant role in particle assembly. Thus, the deletion of up to 26 N-terminal amino acids did not interfere with VLP assembly and did not result in significant changes in their morphology [19]. In addition, the replacement of 44 N-terminal amino acids with epitopes of canine parvovirus and canine distemper virus (maximum insert size up to 68 a.a.) [17] as well as foot-and-mouth disease (FMD) (maximum insert size up to 65 a.a.) [15], did not prevent the formation of VLPs. However, it is obvious that the correct assembly of VLPs is influenced by the specificity of the amino acid sequence of the insert. Thus, the replacement of the first 42 amino acids of the PhMV capsid protein with an equal-sized sequence of the immunodominant epitope of Japanese Encephalitis Virus (JEV) resulted in the expression of an insoluble protein in inclusion bodies. However, after protein isolation from inclusion bodies and its refolding, VLPs were obtained that were morphologically identical to wild-type viral particles [16]. N-terminal inserts in the PhMV CP were used not only to obtain the candidate VLP-based vaccines but also test systems for detecting serum antibodies to various infectious diseases, such as foot-and-mouth disease virus [15] and bursal disease virus [20]. Although chemical cross-linking has been widely used in recent years to modify VLPs [10,21,22], genetic fusion of the antigen with the CP remains simpler and more cost-effective. 

In this study, we used PhMV CP-derived VLPs as a carrier of the influenza A virus antigen, the M2e peptide. M2e, the extracellular domain of the transmembrane protein M2, is highly conserved and may serve as the basis for the development of “universal” influenza vaccines effective against a broad range of influenza A virus strains [23,24]. M2e-specific antibodies, mainly IgG, do not have virus-neutralizing activity but may confer protection through the mechanism of the antibody-dependent cell-mediated cytotoxicity of infected cells, limiting viral replication further and promoting accelerated viral clearance, as well as reducing the severity of disease [25,26].

M2e is a short peptide (23 a.a.), and M2e-specific antibody responses are hardly induced following infection [27]. Nevertheless, the immune response against M2e can be enhanced by its attachment to a highly immunogenic carrier or adjuvant [23,28,29,30]. The most commonly used approach to increase the immunogenicity of M2e is to create VLPs that present this antigen on the surface. Since 1999, many studies have been published on the generation of VLPs carrying M2e, most of which used the CPs of animal viruses (reviewed in [28,29]). However, several examples of the use of plant viruses and VLPs for M2e presentation are also known.

Chimeric tobacco mosaic virus (TMV) particles carrying M2e were obtained via the genetic insertion of M2e into the CP gene. The chimeric TMV-M2e viruses obtained from plants were immunogenic and protected immunized mice from a lethal influenza A challenge [31,32]. The chemical conjugation of TMV virions and M2e expressed in *E. coli* has also been reported [33]. The M2e peptide was inserted at the C-terminus of the CP of the Alternanthera mosaic virus (AltMV). Chimeric CPs expressed in *N. benthamiana* formed rod-shaped VLPs similar to AltMV VLPs [34]. Papaya Mosaic Virus (PapMV), which has rod-shaped virions, has also been used as a carrier of M2e. PapMV CPs carrying an M2e fusion at the C-terminus can be expressed in *E. coli* and assembled into VLPs, but they showed limited immunogenicity and protective activity in mice [35]. A subsequent study reported that the fusion of a shorter M2e-derived peptide (EVETPIRNE) to the N-terminus of PapMV CPs resulted in the formation of highly immunogenic VLPs that provided protection against an influenza challenge; however, the fusion of longer peptides to CPs impaired VLP formation [36]. To solve this problem, Therien et al. [37] enzymatically cross-linked M2e to PapMV CP-derived VLPs; these particles were immunogenic and protected mice from a lethal influenza challenge.

In contrast to plant viruses that form rod-shaped particles, examples of the use of spherical viruses as M2e carriers are very limited. A chimeric Cowpea Mosaic Virus carrying the M2e peptide has been reported [38]; however, the recombinant virus was unstable and reverted to a wild type, and the CPMV/M2e particles were poorly immunogenic and did not exhibit a protective effect.

In this work, we evaluated the possibility of using the PhMV capsid protein to create VLPs displaying the M2e peptide on the surface. We designed fusion proteins comprising four copies of the M2e peptide inserted into the N-terminus of PhMV CPs. The fusion proteins were expressed in *E. coli* and assembled into VLPs. The immunization of mice with M2e-bearing VLPs induced a strong humoral immune response against M2e and protected the immunized mice from a lethal influenza A virus challenge. Thus, the PhMV capsid protein can be used as an M2e carrier for the development of recombinant influenza vaccines.

## 2. Materials and Methods

### 2.1. Design and Construction of Expression Vectors

The gene encoding CP of PhMV (GenBank S97776; [39]) was synthesized. For more efficient expression in *E. coli*, the codons encoding arginine at positions 140, 177, and 179 were replaced by the CGT codon. Cloning of the PhMV CP gene in the pQE30 plasmid (Qiagen, Hilden, Germany) resulted in the expression vector pQE30_PhMV. The gene encoding the truncated CP, lacking the 43 N-terminal amino acids, was obtained by PCR amplification with primers Pr-F N-PhMV (GGG CCC GGC ACC GCT GAA ACC GCA GC) and Pr-R pQE (CAT TAC TGG ATC TAT CAA CAG G) using pQE30_PhMV as a template. By replacing the CP gene in pQE30_PhMV with this fragment, the vector pQE30_PhMVdel was obtained. Both vectors encoded the tag sequence MRGSHHHHHHGSDIGP, followed by the full-size or truncated PhMV CP.

Four tandem copies of the M2e peptide (4M2e) were used as the target influenza antigen. The M2e sequence matched the consensus sequence of human influenza A strains with two cysteine-to-serine substitutions to prevent disulfide bond formation and protein aggregation (SLLTE VETPI RNEWG SRSND SSD, substitutions underlined) [40]. The 4M2e coding sequence was obtained by PCR and cloned into pQE30_PhMV and pQE30_PhMVdel upstream of the CP gene, resulting in the pQE30_4M2eh-PhMV and pQE30_4M2eh-PhMV_del vectors for expression of 4M2e-CP fusions. In addition, the 4M2e sequence flanked by the flexible glycine-serine linkers 19S (GTSGSSGSGSGGSGGGG [41]) was cloned in a similar manner, resulting in the expression vectors pQE30_19s-4M2eh-19s-PhMV and pQE30_19s-4M2eh-19s-PhMV_del.

The amino acid sequences of all recombinant proteins are provided in the Supplementary Materials.

### 2.2. Expression of Recombinant Proteins

For the expression of recombinant proteins, the corresponding vectors were introduced into the *E. coli* strain DLT1270. To obtain starter cultures, individual colonies were inoculated into 5 mL of 2xTY medium (16 g/L Tryptone, 10 g/L Yeast Extract, and 5.0 g/L NaCl) and grown overnight with shaking at 37 °C.

For protein expression, starter cultures were inoculated 1:100 into fresh 2xTY medium and grown with shaking at 20 °C to the mid-log phase (OD_600_~0.6). Expression was induced by adding isopropyl β-D-1-thiogalactopyranoside (IPTG) to 1 mM, and cultures were further grown overnight at 20 °C.

After induction, the cells were collected by centrifugation and resuspended in PBS buffer (10 mM phosphate-buffered saline and 150 mM NaCl, pH 7.4) supplemented with 1 mg/mL lysozyme and then frozen. After disruption of the cells by sonication (Bandelin SONOPULS, Bandelin Electronic GmbH, Berlin, Germany) at an HD 2200 mode, cycle 45%, the cell lysate was centrifuged at 10,000 rpm for 15 min (Centrifuge 5810R, Eppendorf, Hamburg, Germany), thus separating the soluble proteins from the cellular debris and insoluble proteins. The supernatant was used for further purification of the recombinant proteins.

### 2.3. Purification of Virus-like Particles

The first stage of the purification of VLPs was carried out by precipitation with ammonium sulfate. For this purpose, a 30% saturated ammonium sulfate solution was added to the cell lysate, and NaCl was added to a final concentration of 1 M, and the pH was adjusted to 9.0 with NaOH. VLPs were collected by centrifugation at 13,000 rpm for 2 min using a Mikro 20 centrifuge (Hettich, Kirchlengern, Germany) and dissolved in 10 mM PBS buffer. 

VLPs formed by a full-size or truncated PhMV CP without M2e fusions were purified by sucrose–cesium chloride density gradient ultracentrifugation of the cell lysates. The discontinuous gradient consisted of 2 mL of 1.5 g/L CsCl at the bottom of the tube, followed by 3 mL of 1.2 g/L CsCl, 3 mL of 30% (*w*/*v*) sucrose, and 2 mL of 20% (*w*/*v*) sucrose solution. The partly purified VLP solution was loaded at the top of the tube. Ultracentrifugation was performed in an SW40 rotor in an Optima L-90K Ultracentrifuge (Beckman Coulter, Brea, CA, USA) at 35,000 rpm at 20 °C for 22 h. The resulting gradient was fractionated into 1 mL aliquots and then analyzed by SDS-PAGE. 

For purification of the 19s-4M2eh-19s-PhMV VLPs, the second step was metal-affinity chromatography on Ni-NTA-agarose (Qiagen, Hilden, Germany). The recombinant protein was adsorbed onto agarose in 10 mM PBS at pH 7.4 with 1.15 mM NaCl and 1 M urea. Unbound proteins were then washed out with the same buffer, which also contained 16 mM imidazole. Adsorbed proteins were eluted with 10 mM PBS containing 250 mM imidazole. After purification, the proteins were dialyzed against 10 mM PBS with 150 mM NaCl.

### 2.4. Structural Analysis of VLPs

The presence of virus-like particles was confirmed by dynamic light scattering and electron microscopy. Electron microscopy was performed on a JEM 1400 instrument (JEOL, Tokyo, Japan). Purified proteins were loaded onto carbon–formvar-coated copper grids (TED PELLA, Redding, CA, USA) and stained with 1% (*w*/*v*) uranyl acetate in methanol. Analysis of the particle sizes by a dynamic light scattering method was performed at 37 °C using a Zetasizer NanoS90 particle size analyzer (Malvern Panalytical, Malvern, UK) with 12 mm square polystyrene cuvettes (DTS0012). The polydispersity index (PDI) was calculated as the square of the polydispersity (standard deviation/mean). 

### 2.5. Recognition of M2e-Carrying VLPs by Anti-M2e Antibodies in ELISA

Two-fold dilutions of purified PhMV and 19s-4M2eh-19s-PhMV proteins (starting at 4 μg/mL) in a sodium bicarbonate buffer (pH 8.5) were loaded into wells of ELISA plates (Greiner, Kremsmünster, Austria) and incubated overnight at 4 °C. The plates were washed with PBST (PBS with 0.05% Tween) and then incubated with blocking buffer (0.2% (*w*/*v*) BSA in PBS) for 1 h at 37 °C. After a single wash with PBS, the plates were probed with home-made mouse polyclonal antibodies specific for M2e (at 1:20,000 dilution) for 30 min at 37 °C. After five washes with PBST, a solution of the peroxidase-labeled anti-mouse IgG antibody (at 1:10,000 dilution) was added. The plates were then incubated with the conjugate for 30 min and washed five times with PBST. Tetramethylbenzidine (Vector-BEST, Novosibirsk, Russia) was used as a substrate for horseradish peroxidase. The reaction was stopped by adding HCI (0.5 N), and the optical density was measured at 450 nm using a microplate spectrophotometer. 

### 2.6. Immunization of Mice

The study used female *BALB/c* mice (haplotype H-2d) weighing 17–19 g (15 per group). The animals were subcutaneously immunized with PhMV and 19s-4M2eh-19s-PhMV particles three times, with a two-week interval at a dose of 50 μg/mouse (80 μL) without adjuvants. Mice in the control group were likewise administered PBS.

### 2.7. Measurement of Antibody Titers in Sera by ELISA

Serum samples were obtained from five mice in each group two weeks after the third immunization. The sera were analyzed by ELISA using 96-well plates (Greiner, Frickenhausen, Germany). Synthetic peptide G37 corresponding to the consensus sequence of M2e from human influenza A strains (SLLTEVETPIRNEWGCRCNDSSD) at a concentration of 5 μg/mL was immobilized on the plate. Polyclonal goat anti-mouse IgG (Abcam, Cambridge, UK) labeled with horseradish peroxidase (at 1:5000 dilution) was used as a secondary antibody. The complexes were revealed by adding a 3,3,′5,5′-tetramethylbenzidine (Biolegend, San Diego, CA, USA) substrate solution for 15 min. The reaction was stopped by the addition of H_2_SO_4_. Absorption was measured using an ELISA Reader at 450 nm.

### 2.8. Influenza Virus and Challenge Experiment

To assess the protective properties of the studied VLPs, a mouse-adapted influenza strain A/Aichi/2/68 (H3N2) was used at a calculated dose of 5 LD_50_. The strain was obtained from the Collection of Influenza and Acute Respiratory Viruses at the Research Institute of Influenza. The virus was administered intranasally two weeks after the third immunization. The survival and weight of mice (10 per group) after the challenge were recorded daily for 14 days. 

### 2.9. Statistical Analysis

Statistical analysis was performed using GraphPad Prism v. 10.2.2. To compare the groups, one-way ANOVA with Tukey’s multiple comparisons test was used. Significant differences in survival between the mouse groups were analyzed using the Mantel–Cox test. Differences were considered significant at *p* < 0.05.

### 2.10. Ethics Statement

The study was conducted in accordance with the recommendation of the Board of the Eurasian Economic Commission “On the Guidelines for working with laboratory (experimental) animals when conducting preclinical (non-clinical) studies” (14 November 2023, No. 231 33). The experiments were approved by the Bioethics Committee on the Use of Animals of the Smorodintsev Research Institute of Influenza (Permit ID No. 24a/08/23). All possible efforts were made to minimize animal suffering. 

## 3. Results

### 3.1. Design and Construction of Recombinant Proteins and Expression Vectors

**Figure 1:**
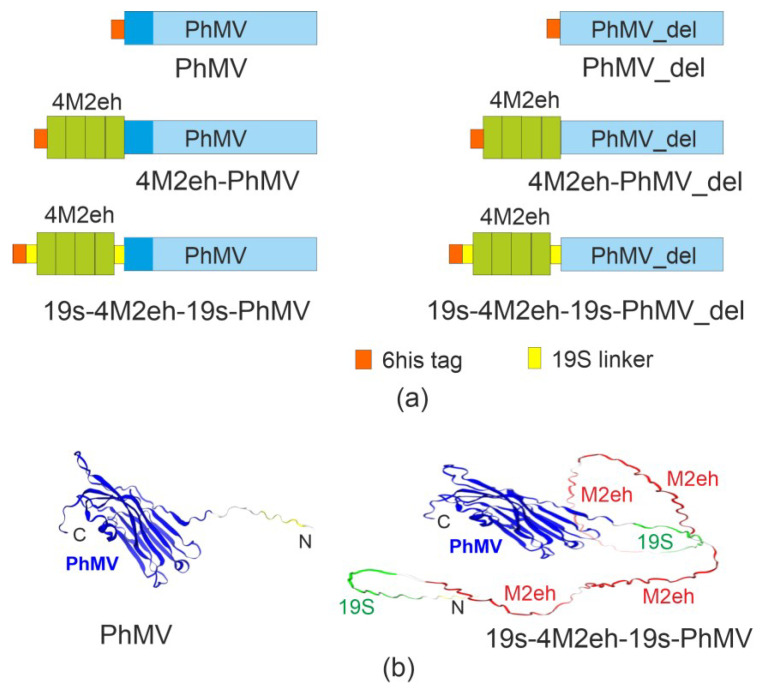
(**a**) Schematic representation of the recombinant protein structures. 4M2eh, four tandem copies of the M2e peptide; PhMV, the PhMV coat protein; PhMV_del, a truncated coat protein of PhMV with the deletion of the N-terminal region (shown in dark blue in PhMV). (**b**) 3D modeling of the structures of PhMV CP and 19s-4M2eh-19s-PhMV proteins using Alphafold v.2.3.1 [43]. Visualization was performed using the SWISS MODEL server [44]. The N- and C- termini are shown.

The M2e peptide whose sequence matched the consensus sequence of human influenza A strains was used to develop a new influenza vaccine candidate. Since increasing the number of M2e copies fused to the capsid proteins forming VLPs from one to four enhanced the immune response [42], four consecutive copies of M2e were fused to the PhMV CP. Since previous studies have shown that the N-terminus of the CP can be used for modifications, and the intactness of the C-terminus is critical for VLP assembly [19], 4M2e inserts were attached to the N-terminus of the PhMV CP. The results of the CP structure modeling also suggest a free position of the N-terminus of the molecule (Figure 1).

In addition to the full-length CP, a truncated CP lacking the first 43 amino acids was used as a building block for VLP assembly. Cloning of the hybrid genes in the pQE30 expression vector resulted in the creation of pQE30_4M2eh-PhMV and pQE30_4M2eh-PhMV_del vectors for the expression of 4M2e fused to the full-length and truncated PhMV capsid, respectively (Figure 1). Empty capsid proteins without the 4M2e insert were expressed using the pQE30_PhMV and pQE30_PhMV_del vectors. The hexahistidine tag encoded by the pQE30 vector was fused to the N-terminus of the recombinant proteins to allow their purification by metal affinity chromatography.

Since the insertion of the 4M2e peptide may change the conformation of the recombinant protein, we introduced a glycine-serine-rich 19S linker between 4M2e and the PhMV CP to facilitate the folding of the fusion proteins and efficient presentation of the target antigen on the VLP surface. The same linker was inserted between the 4M2e peptide and the N-terminal hexahistidine tag to facilitate the interaction of the proteins with the Ni-NTA sorbent. The constructed vectors pQE30_19s-4M2eh-19s-PhMV and pQE30_19s-4M2eh-19s-PhMV_del allowed the expression of these variants of the fusion proteins (Figure 1).

**Figure 2:**
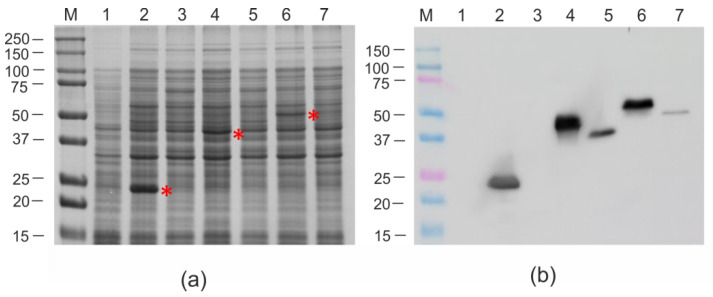
Expression of recombinant proteins in *E.coli*. Proteins isolated from *E.coli* were analyzed by SDS-PAGE (**a**) or Western blotting with antibodies against the hexahistidine tag (**b**). M, molecular weight marker (sizes are shown in kD). Total proteins isolated from the *E. coli* strain without expression vector (lane 1); total proteins isolated after induction from *E. coli* strains carrying plasmids pQE30_PhMV (lane 2), pQE30_PhMV_del (lane 3), pQE30_4M2eh-PhMV (lane 4), pQE30_4M2eh-PhMV_del (lane 5), pQE30_19s-4M2eh-19s-PhMV (lane 6), and pQE30_19s-4M2eh-19s-PhMV_del (lane 7). The positions of recombinant proteins in SDS-PAGE are marked with red asterisks.

The fusion genes cloned into pQE30 were expressed in *E. coli*. At temperatures above 28 °C after the induction of expression, all recombinant proteins were insoluble. The maximum production of soluble target proteins was observed when the culture was grown in a rich 2xTY medium at 20 °C (Figure 2). The recombinant proteins PhMV, 4M2eh-PhMV, and 19s-4M2eh-19s-PhMV were expressed at a level of about 5–15% of the total protein (Figure 2). In contrast, the truncated variant of the PhMV CP, as well as its fusions with 4M2e, were poorly expressed (Figure 2). Therefore, the full-length PhMV capsid protein and the 19s-4M2eh-19s-PhMV protein with the 4M2eh insert flanked by glycine-rich linkers, which provide better accessibility of the histidine tag, were selected for further work.

### 3.2. Purification of VLPs

The VLPs formed by the recombinant proteins were purified in two steps. First, the partial purification and concentration of the VLPs were performed by adding a 30% saturated ammonium sulfate solution at a high concentration of NaCl. The second purification step was different for the two recombinant proteins.

**Figure 3:**
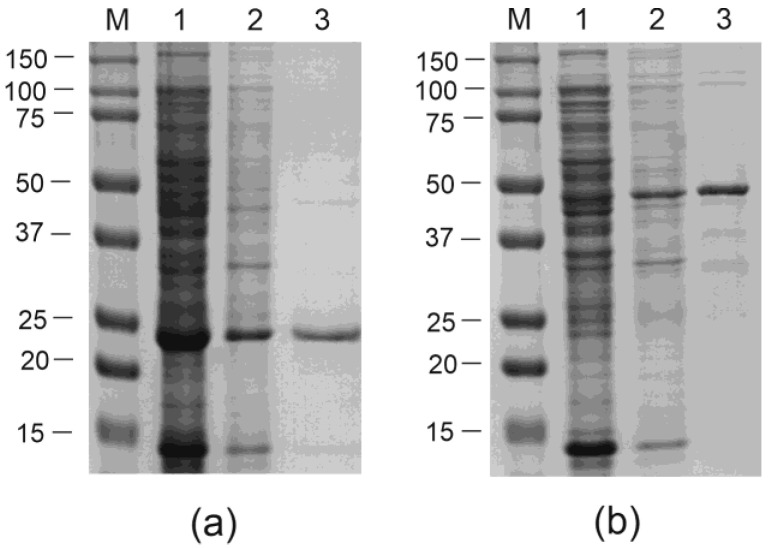
Purification of VLPs formed by PhMV (**a**) and 19s-4M2eh-19s-PhMV (**b**) proteins. The proteins were analyzed by SDS–PAGE. M, molecular weight marker (sizes are shown in kD). Lanes: 1,—clarified cell lysate; 2,—VLPs after the first step of purification (precipitation with ammonium sulfate); 3,—VLPs after the second step of purification using ultracentrifugation (**a**) or nickel affinity chromatography (**b**).

VLPs formed by the empty CPs of PhMV were purified by ultracentrifugation in a sucrose–cesium chloride gradient (Figure 3).

Particles bearing the M2e peptide were purified by nickel affinity chromatography (Figure 3). For the efficient purification of the 19s-4M2eh-19s-PhMV protein, the concentrations of NaCl and urea were increased in the adsorption and washing solutions. Increasing the NaCl concentration to 1.15 M reduced the content of non-target proteins, which probably adhered to the particles due to electrostatic interactions, in the purified VLP preparation. Increasing the urea concentration to 1 M enabled better accessibility of the hexahistidine tag for the sorbent without destroying the VLP. It is known that native PhMV VLPs are highly stable and are not destroyed in solutions containing up to 4 M urea [19].

**Figure 4:**
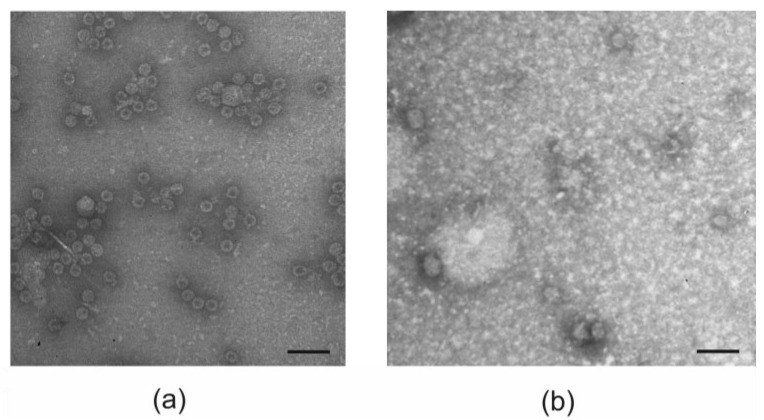
Analysis of the structure of VLPs formed by PhMV (**a**) and 19s-4M2eh-19s-PhMV (**b**) proteins using electron microscopy. Scale bar, 100 nm.

The assembly of purified recombinant proteins into nanosized particles was examined using dynamic light scattering and electron microscopy. Electron microscopy analysis shows that both the empty PhMV capsids and 19s-4M2eh-19s-PhMV proteins formed spherical particles of approximately 30 nm in size (Figure 4). In the case of the 19s-4M2eh-19s-PhMV protein, along with spherical particles, elongated structures were also observed (Figure 4).

The particle size of the VLP estimated using a dynamic light scattering approach was about 40 nm (Table 1). The PhMV protein also formed larger structures of 100–200 nm in size (Supplemental File S2). The data on the polydispersity index (PDI) indicate that 19s-4M2eh-19s-PhMV particles are more heterogeneous in size than those formed by empty PhMV capsids (Table 1).

### 3.3. Antigenic Properties of 4M2e-Carrying Chimeric VLPs

**Figure 5:**
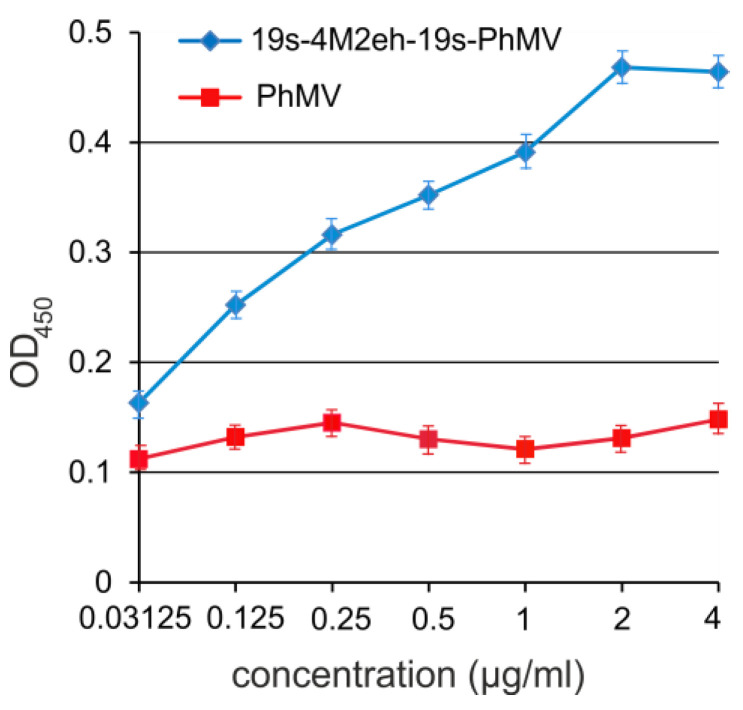
Antigenic properties of VLPs. Two-fold dilutions of VLPs formed by PhMV and 19s-4M2eh-19s-PhMV proteins were loaded onto ELISA plates and then probed with anti-M2e antibodies.

Computational modeling of the PhMV and 19s-4M2eh-19s-PhMV protein structures predicted a free spatial location of the 4M2e peptide at the N-terminus of the CP relative to the rest of the fusion protein (Figure 1b). Therefore, the exposure of the 4M2e peptides on the surface of the assembled VLP and their accessibility to specific antibodies could be expected. Purified VLPs were immobilized on ELISA plates under native conditions and probed with M2e-specific monoclonal antibodies (Figure 5).

The results show that 19s-4M2eh-19s-PhMV particles efficiently bind anti-M2e antibodies, unlike the control PhMV particles. Since the VLPs were not deconstructed before loading to the plate, the obtained data suggest that the M2e peptide is exposed on the particle surface.

### 3.4. Immunogenicity and Protective Activity of 4M2e-Carrying Chimeric VLPs

**Figure 6:**
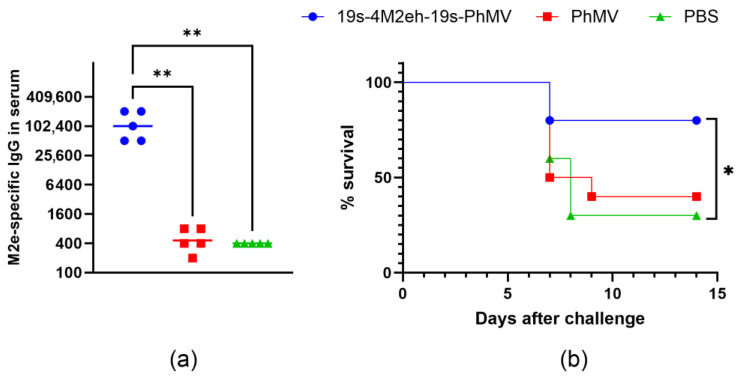
Immunogenicity and protective efficiency of VLPs. (**a**) Titers of anti-M2e IgG in sera of immunized mice. Data are presented as geometric mean titers (GMTs) and values observed in individual mice. (**b**) Protective efficacy of recombinant proteins. Statistically significant differences between groups are indicated (**, *p* < 0.01; *, *p* < 0.05).

To evaluate the immunogenicity of chimeric VLPs, *BALB/c* mice were immunized three times at 2-week intervals with 19s-4M2eh-19s-PhMV particles. PhMV particles without M2e fusion and PBS were administered as controls. After the third immunization, the blood sera were tested for the presence of anti-M2e IgG antibodies using ELISA. Mice immunized with 19s-4M2eh-19s-PhMV particles developed a strong M2e-specific immune response, with antibody titers reaching 100,000 (Figure 6a). The titers were significantly higher compared to the control groups immunized with “empty” PhMV particles and PBS (*p* < 0.01).

To assess the protective efficacy of 19s-4M2eh-19s-PhMV particles, immunized and PBS control mice were challenged with an influenza A/Aichi/2/68 (H3N2) strain. The survival of mice was observed for 14 days. The immunization of mice with 4M2e-carrying VLPs resulted in 80% protection, while the survival rate in the control groups was 30–40% (Figure 6b). The differences in survival between mice immunized with 19s-4M2eh-19s-PhMV particles and the control mice treated with PBS were statistically significant (*p* = 0.0389). The severity of the disease was monitored by measuring the weight of the mice, but no difference was observed between the three groups (Supplemental File S3).

## 4. Discussion

Due to the spread and emergence of new infectious pathogens, the search for new vaccine platforms is an important task. One of the most promising approaches in this area is the search for new antigen carriers based on virus-like particles. Most often, the capsid proteins of animal viruses are used as the basis for such VLPs, but plant viruses are an attractive alternative. One concern with using VLPs derived from animal viruses may be that pre-existing immunity from vaccination or previous infection may make vaccination using the same VLP carrier less effective. VLPs based on plant viruses are inherently safe, and most importantly, the likelihood of a person having antibodies to them is minimal. At the same time, they retain all the advantages of a carrier VLP. This includes a strictly symmetrical structure, the ability to present multiple copies of antigen on its surface, and a small size. The nanoscale size of VLPs ensures the efficient transport of the target antigen to immune cells due to the possibility of the free drainage of the lymph nodes [45,46].

We chose the PhMV capsid protein as the vaccine carrier. This is one of the few plant viruses with a spherical virion, which has been used as an antigen carrier. Unlike viruses with helical symmetry, the virions of spherical viruses have a more rigid composition and structure. When produced in other expression systems, the recombinant capsids of spherical viruses often retain their original shape and structure [6,7]. Moreover, PhMV-based VLPs have already been used as carriers of heterogeneous antigens [15] and have shown adjuvant properties [10,17,22].

We used the external domain of the M2 protein as an antigen to develop a recombinant influenza vaccine. The consensus sequence of the M2e peptide of the human influenza A virus was modified by replacing two cysteines with serines to prevent protein aggregation due to the formation of disulfide bridges. As shown previously, this modification does not lead to a change in the immunogenicity of M2e [40]. We also increased the number of copies of the M2e peptide in the fusion protein to four, as this has been repeatedly shown to increase immunogenicity [42,47,48]. In addition, 19s glycine-rich linkers were inserted on both sides of 4M2e. The presence of linkers provided flexibility in the structure of the recombinant protein, ensuring better separation from the VLP core of both the antigen insert and the hexahistidine tag necessary for protein purification.

Previous studies have shown that the deletion of up to 26 N-terminal a.a. of the PhMV CP or the insertion of foreign peptides up to 68 a.a. in length does not interfere with VLP assembly [15,17]. However, in our study, the insert size was significantly larger at 154 a.a. (including linkers and the hexahistidine tag), which is comparable to the size of the PhMV capsid protein itself (188 a.a.). Thus, in addition to fusing antigens to the N-terminus of the full-length capsid protein, we also obtained similar hybrid proteins based on a truncated CP lacking the first 43 a.a., as described previously [17].

Quite unexpectedly, the expression levels in *E. coli* cells of both the truncated CP and fusions based on it were many times lower than those of the full-length PhMV CP and similar fusion proteins based on it (Figure 2). The reasons for this are unclear. Structural modeling did not reveal any differences in the spatial structures of the full-length and truncated CP, except for the absence of a protruding N-terminal tail in the latter. Therefore, recombinant proteins based on the full-length capsid were used for further experiments.

Interestingly, according to both electron microscopy and DLS data, the PhMV CP with the insert formed particles of nearly the same size as the empty capsid. The particles with M2e peptides were more heterogeneous in size. It is possible that the PhMV CP forms a hard particle core, with the inserted peptides forming a less dense, undetectable coating. Similar patterns have previously been observed for HBc and phage capsid-based VLPs carrying four copies of M2e [42,49].

The purification of recombinant VLPs was performed in different ways for VLPs with and without antigen inserts. Particles without inserts were purified by ultracentrifugation in a sucrose–cesium chloride density gradient. Particles with inserts were also subjected to this procedure. However, contaminating proteins were observed in the fractions with VLPs. This was probably due to the nonspecific binding of bacterial proteins to the 4M2e tails due to electrostatic interactions. The purification of 19s-4M2eh-PhMV particles by affinity chromatography on a Ni-NTA sorbent under standard conditions was also unsuccessful because the recombinant protein, upon elution, appeared to be contaminated by non-target bacterial proteins. However, the purified 19s-4M2eh-PhMV particles were obtained by increasing the salt concentration during affinity chromatography. The accessibility of the 6-histidine tag was facilitated by the addition of urea. The unique nature of PhMV particles makes them extremely resistant to denaturing agents, and recombinant PhMV VLPs were not destroyed even by 4 M urea [19].

The animal immunization experiments demonstrate the induction of high levels of M2e-specific antibodies, consistent with the localization of the 4M2e peptide on the surface of 19s-4M2eh-19s-PhMV particles predicted by the analysis of their antigenic properties. Anti-M2e antibody titers reached approximately 100,000, which is comparable to the values obtained when mice were immunized with other M2e-carrying nanoparticles [42,49,50]. Despite high immunogenicity, the chimeric 19s-4M2eh-19s-PhMV particles conferred only incomplete (80%) protection of animals from infection with a lethal dose of influenza A/Aichi/2/68 (H3N2) virus and did not reduce the severity of the disease, assessed by weight loss. Therefore, further optimization of the M2e-based vaccine candidate will be required, such as adding conserved epitopes from the hemagglutinin and/or nucleoprotein to the PhMV-based VLP.

## 5. Conclusions

The full-length PhMV CP can be used to generate chimeric VLPs carrying large peptides, the size of which is comparable to the size of the CP itself. A PhMV CP carrying 4 copies of the M2e peptide of the influenza A virus at the C-terminus could be efficiently expressed in *E. coli* cells and assembled into spherical VLPs resembling PhMV virions. M2e peptides were displayed on the surface of the VLPs and were recognized by anti-M2e antibodies. The immunization of mice with these recombinant VLPs induced high levels of M2e-specific IgG antibodies in sera and protected mice from a lethal influenza A virus challenge. Overall, PhMV particles carrying M2e peptides can be used to develop a recombinant influenza vaccine.

## Figures and Tables

**Table 1:** VLPs characteristics determined using dynamic light scattering.

Parameter	PhMV	19s-4M2eh-19s-PhMV
Size (nm) *	42.1 ± 3.4	37.5 ± 1.8
PDI	0.24	0.41

* Mean ± standard deviation.

## Data Availability

The data presented in this study are contained within the article.

## Supplementary Materials

The following supporting information can be downloaded at: https://www.mdpi.com/article/10.3390/v16111802/s1, File S1: Amino acid sequences of recombinant proteins; File S2: Analysis of VLPs using dynamic light scattering; File S3: Changes in weight of immunized mice after infection.
