# Supplementary material for: Chimeric Virus-like Particles of Physalis Mottle Virus as Carriers of M2e Peptides of Influenza a Virus
Source: Viruses. 2024 Nov 20;16(11):1802. doi: 10.3390/v16111802 (PMC11598990; doi:10.3390/v16111802)
Supplement: Supplementary file 1 [file viruses-16-01802-s001.zip › viruses-3242462-supplementary/File S1.pdf]

Amino acid sequences of recombinant proteins

Color codes:

CP of PhMV (N-terminal a.a. deleted in PhMV_del proteins are underlined

M2e peptide

GS-rich linker

PhMV

MRGSHHHHHHGSDIGPDSSEVVKVKQASIPAPGSILSQPNTEQSPAIVLPFQFEATTFGTAETAAQVSLQTADPITKLTAPYRHAQIVECKAILTPTDLAVSNPLTVYLAWVPANSPATPTQILRVYGGQSFVLGGAISAAKTIEVPLNLDSVNRMLKDSVTYTDTPKLLAYSRAPTNPSKIPTASIQISGRIRLSKPMLIAN

4M2eh-PhMV

MRGSHHHHHHGSDLSLLTEVETPIRNEWGSRSNDSSDDLSLLTEVETPIRNEWGSRSNDSSDDLSLLTEVETPIRNEWGSRSNDSSDDLSLLTEVETPIRNEWGSRSNDSSDDIGPDSSEVVKVKQASIPAPGSILSQPNTEQSPAIVLPFQFEATTFGTAETAAQVSLQTADPITKLTAPYRHAQIVECKAILTPTDLAVSNPLTVYLAWVPANSPATPTQILRVYGGQSFVLGGAISAAKTIEVPLNLDSVNRMLKDSVTYTDTPKLLAYSRAPTNPSKIPTASIQISGRIRLSKPMLIAN

19s-4M2eh-19s-PhMV

MRGSHHHHHHGSGTSGSSGSGSGGSGSGGGGELSLLTEVETPIRNEWGSRSNDSSDDLSLLTEVETPIRNEWGSRSNDSSDDLSLLTEVETPIRNEWGSRSNDSSDDLSLLTEVETPIRNEWGSRSNDSSDDIGTSGSSGSGSGGSGSGGGGGPDSSEVVKVKQASIPAPGSILSQPNTEQSPAIVLPFQFEATTFGTAETAAQVSLQTADPITKLTAPYRHAQIVECKAILTPTDLAVSNPLTVYLAWVPANSPATPTQILRVYGGQSFVLGGAISAAKTIEVPLNLDSVNRMLKDSVTYTDTPKLLAYSRAPTNPSKIPTASIQISGRIRLSKPMLIAN

PhMV_del

MRGSHHHHHHGSDIGPGTAETAAQVSLQTADPITKLTAPYRHAQIVECKAILTPTDLAVSNPLTVYLAWVPANSPATPTQILRVYGGQSFVLGGAISAAKTIEVPLNLDSVNRMLKDSVTYTDTPKLLAYSRAPTNPSKIPTASIQISGRIRLSKPMLIAN

4M2eh-PhMV_del

MRGSHHHHHHGSDLSLLTEVETPIRNEWGSRSNDSSDDLSLLTEVETPIRNEWGSRSNDSSDDLSLLTEVETPIRNEWGSRSNDSSDDLSLLTEVETPIRNEWGSRSNDSSDDIGPGTAETAAQVSLQTADPITKLTAPYRHAQIVECKAILTPTDLAVSNPLTVYLAWVPANSPATPTQILRVYGGQSFVLGGAISAAKTIEVPLNLDSVNRMLKDSVTYTDTPKLLAYSRAPTNPSKIPTASIQISGRIRLSKPMLIAN

19s-4M2eh-19s-PhMV_del

MRGSHHHHHHGSGTSGSSGSGSGGSGSGGGGELSLLTEVETPIRNEWGSRSNDSSDDLSLLTE
VETPIRNEWGSRSNDSSDDLSLLTEVETPIRNEWGSRSNDSSDDLSLLTEVETPIRNEWGSRSN
DSSDDIGTSGSSGSGSGGSGSGGGGGPGTAETAAQVSLQTADPITKLTAPYRHAQIVECKAILT
PTDLAVSNPLTVYLAWVPANSPATPTQILRVYGGQSFVLGGAISAAKTIEVPLNLDSVNRMLK
DSVTYTDTPKLLAYSRAPTNPSKIPTASIQISGRIRLSKPMLIAN
