# Supplementary material for: Chimeric Virus-like Particles of Physalis Mottle Virus as Carriers of M2e Peptides of Influenza a Virus
Source: Viruses. 2024 Nov 20;16(11):1802. doi: 10.3390/v16111802 (PMC11598990; doi:10.3390/v16111802)

Analysis of VLPs using dynamic light scattering

PhMV particles

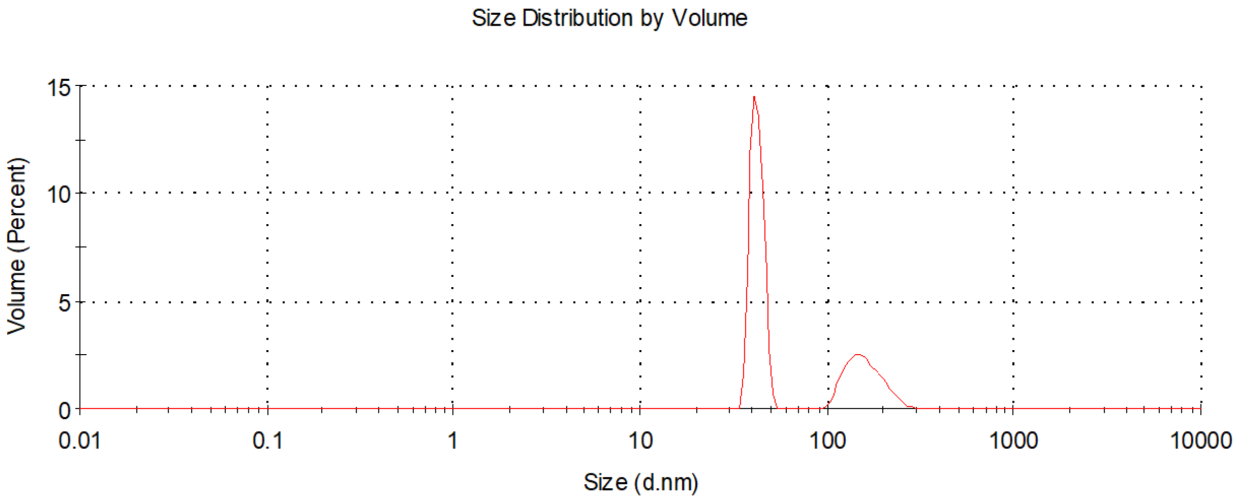

19s-4M2eh-19s-PhMV particles

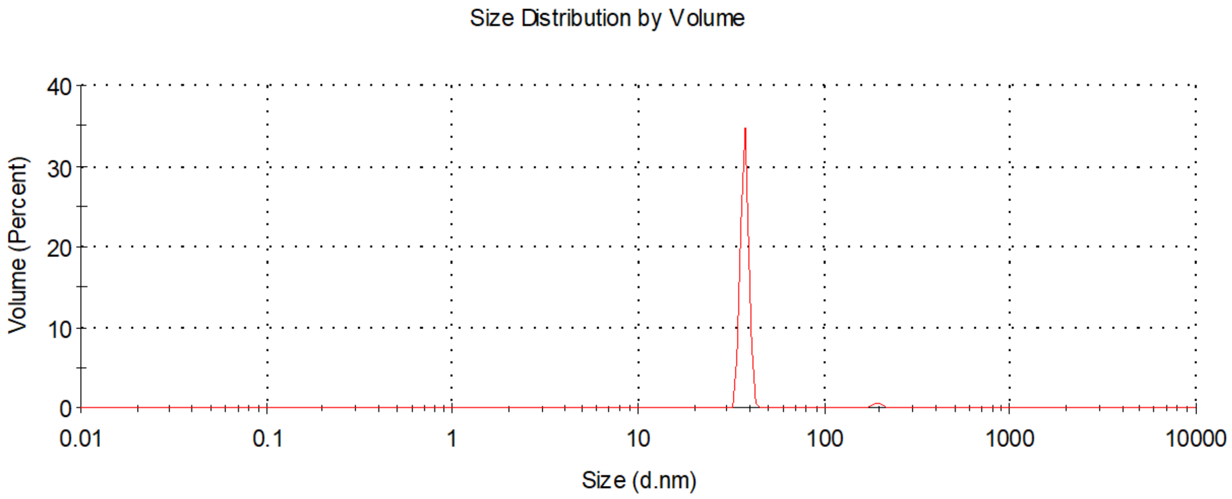

Supplement: Supplementary file 1 [file viruses-16-01802-s001.zip › viruses-3242462-supplementary/File S2.pdf]
