# Supplementary material for: Chimeric Virus-like Particles of Physalis Mottle Virus as Carriers of M2e Peptides of Influenza a Virus
Source: Viruses. 2024 Nov 20;16(11):1802. doi: 10.3390/v16111802 (PMC11598990; doi:10.3390/v16111802)
Supplement: Supplementary file 1 [file viruses-16-01802-s001.zip › viruses-3242462-supplementary/File S3.pdf]

Changes in weight of immunized mice after infection

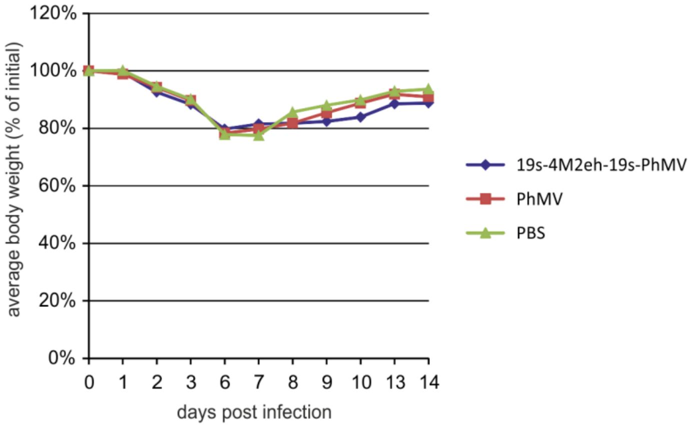

Body weight of immunized mice was monitored for 14 days post-challenge. The average change in animal weight for each group is shown.
